# Liver transplantation for very severe hepatopulmonary syndrome due to vitamin A-induced chronic liver disease in a patient with Shwachman-Diamond syndrome

**DOI:** 10.1186/s13023-018-0818-2

**Published:** 2018-05-02

**Authors:** Giorgia Bucciol, David Cassiman, Tania Roskams, Marleen Renard, Ilse Hoffman, Peter Witters, Rik Schrijvers, Heidi Schaballie, Barbara Bosch, Maria Caterina Putti, Olivier Gheysens, Noel Knops, Marc Gewillig, Djalila Mekahli, Jacques Pirenne, Isabelle Meyts

**Affiliations:** 1 Laboratory of Inborn Errors of Immunity, Department of Microbiology and Immunology, KU Leuven, Herestraat 49, 3000 Leuven, Belgium; 2 Pediatric Oncology and Hematology, Department of Child’s and Woman’s Health, University of Padova, Padova, Italy; 3 Gastroenterology and Hepatology, University Hospitals Leuven and KU Leuven, Leuven, Belgium; 4 Anatomic Pathology, University Hospitals Leuven and KU Leuven, Leuven, Belgium; 5 Department of Pediatrics, University Hospitals Leuven, Leuven, Belgium; 6 Laboratory of Clinical Immunology, Microbiology and Immunology Department, University Hospitals Leuven and KU Leuven, Leuven, Belgium; 7 St. Giles Laboratory of Human Genetics of Infectious Disease, Rockefeller Branch, The Rockefeller University, New York, USA; 8 Nuclear Medicine, University Hospitals Leuven and KU Leuven, Leuven, Belgium; 9 Department of Cardiovascular Sciences, KU Leuven, Leuven, Belgium; 10 Department of Pediatric Cardiology, University Hospitals Leuven, Leuven, Belgium; 11 Department of Development and Regeneration, KU Leuven, Leuven, Belgium; 12 Department of Pediatric Nephrology, University Hospitals Leuven, Leuven, Belgium; 13 Abdominal Transplantation Surgery, Laboratory of Abdominal Transplantation, University Hospitals Leuven and KU Leuven, Leuven, Belgium

**Keywords:** Vitamin A, Liver transplantation, Shwachman-Diamond syndrome, Hepatopulmonary syndrome

## Abstract

Vitamin A intoxication is a rare cause of liver disease, but the risk increases in patients with underlying liver dysfunction. We present a patient with Shwachman-Diamond Syndrome who developed liver fibrosis, portal hypertension and very severe hepatopulmonary syndrome as a consequence of chronic vitamin A intoxication. She underwent successful liver transplantation with complete resolution of the pulmonary shunting.

## Introduction

Shwachman-Diamond syndrome (SDS) is a congenital ribosomopathy caused by biallelic mutations in the *Shwachman-Bodian-Diamond syndrome* (*SBDS*) gene. SDS is characterized by exocrine pancreatic insufficiency, bone marrow failure and metaphyseal chondrodysplasia. The typical presentation occurs in infancy with failure to thrive, metaphyseal dysplasia and neutropenia. Anemia and thrombocytopenia develop in almost 50% of patients. Immunodeficiency is evident in most patients [1, 2]. Moreover, the risk of myelodysplastic syndrome (MDS) and myeloid leukemia is higher than the general population [1, 2]. Hepatomegaly with elevated transaminases is present in 75% of patients with SDS under 5 years of age [2, 3]. Treatment comprises pancreatic enzymes substitution, granulocyte colony stimulating factor (G-CSF) and antibiotics for symptomatic neutropenia. Due to the exocrine pancreatic insufficiency, patients with SDS typically receive supplements of vitamin A, D, E and K.

Vitamin A excess is toxic. Its biological half-life is 286 days and it accumulates in various tissues, especially in the liver. Symptoms of vitamin A intoxication include anorexia, fatigue, hepatomegaly, alopecia, skin desquamation, cheilitis, bone pain, bulging fontanels, craniotabes, pseudotumor cerebri, photophobia and hypoplastic anemia [4]. Chronic vitamin A intoxication causes liver fibrosis and portal hypertension, especially in patients with underlying congenital or acquired liver disease [5].

Hepatopulmonary syndrome (HPS) is a common vascular complication of liver disease, characterized by intrapulmonary vasodilation (IPVD) and gas exchange anomalies resulting in hypoxemia [6]. Its manifestations are progressive dyspnea and cyanosis, digital clubbing, spider naevi, orthodeoxia (desaturation upon standing from a reclined position) and platypnea (relieving of dyspnea when assuming a reclining position). Diagnosis is based on the demonstration of IPVD and hypoxemia in a patient with liver disease, by using either contrast echocardiography or nuclear lung scanning (99 m-Technetium-labelled macro-aggregated albumin scan – MAA scan), the latter if intracardiac shunt has been excluded [6]. The only available treatment for HPS is liver transplantation, after which hypoxemia generally resolves within one year. It is reported that survival after transplantation is similar for patients with HPS and the general cohort of liver transplant patients. However, in patients with advanced stages of HPS (defined as PaO2 < 50 mmHg on blood gas analysis and/or a shunt fraction on nuclear lung scanning > 20%) it has been shown that complication rate and mortality connected to hypoxia after transplantation are increased [6]. Despite this, it has been recently recommended by the International Liver Transplant Society that severe/very severe HPS should be an indication for expedited liver transplantation, and our personal observations and more recent reports confirm a good outcome in these cases [6, 7].

Here we describe a 15-year-old patient with SDS who developed liver fibrosis and portal hypertension as a consequence of chronic vitamin A intoxication. She presented with cyanosis, which was caused by very severe HPS. Despite the underlying condition and the predicted high complication rate due to the high MAA shunt fraction, she underwent deceased-donor orthotopic liver transplantation without major complications and with complete resolution of the pulmonary shunting. This patient’s SDS phenotype was previously described as patient 6 in a Belgian SDS cohort [8].

## Case report

A 2-year-old girl with failure to thrive, recurrent respiratory and skin infections and pancreatic insufficiency was clinically diagnosed with SDS. Sanger sequencing of *SDBS* showed homozygosity for *c258 + 2 T > C*. She was treated with pancreatic enzyme replacement, vitamin A-D-E-K, zinc and tube feeding for several years for severe malnutrition. A liver biopsy performed at age 2 years in the context of persistently elevated transaminases and intermittently increased bilirubin showed mild periportal fibrosis and chronic cholestasis, which were attributed to the underlying condition SDS. Blood vitamin A, E, D levels and prothrombin time were frequently monitored, and vitamin A levels remained always below the reference range of 30 − 65 μg/dL. For this reason, vitamin A supplementation was progressively increased over time from around 3000 IU per day (derived from vitamin supplements and tube feeding) at the age of 2 years, to over 100,000 IU per day at the age of 11 years.

At age 12 years the patient developed splenomegaly (spleen was at 7 cm from the costal margin on examination) with signs of hypersplenism. Upon physical examination she presented dry skin, cheilitis, hair loss and persistent dystrophic appearance. Blood tests showed mild anemia, neutropenia and thrombocytopenia, prolonged prothrombin time and slightly elevated transaminases (Table 1). Vitamin A levels at this time were still within the reference range. A second liver biopsy at this time demonstrated septal fibrosis and hyperplasia of hepatic stellate cells (i.e. perisinusoidal Ito cells), which on electron microscopy were enlarged and filled with abnormally large and numerous fat drops, suggestive of vitamin A intoxication. Vitamin A supplementation was discontinued and treatment with ursodeoxycholic acid started *ex juvantibus*. Vitamin A blood levels subsequently decreased from normal values (36 μg/dL at the time of biopsy) to very low values (< 10 μg/dL). Nevertheless portal hypertension with gastropathy and esophageal varices ensued. All other cases of advanced liver disease were excluded.

**Table 1:** Clinical and laboratory characteristics of the patient at diagnosis of SDS (2 years), diagnosis of liver disease (12 years), diagnosis of HPS (15 years), liver transplantation (18 years) and at the present follow-up

	Age 2	Age 12	Age 15	Age 18 Liver transplant	Age 22
Height	74.6 cm (< 3° %ile)	129 cm (< 3° %ile)	135 cm (< 3° %ile)	140 cm (< 3° %ile)	141 cm (< 3° %ile)
Weight	7.6 kg (< 3° %ile)	21 kg (< 3° %ile)	30 kg (< 3° %ile)	41 kg (< 3° %ile)	44.2 kg (< 3° %ile)
CMV-PCR	NA	Negative	Negative	Negative	Negative
SatO2 at rest	100%	99%	88%	79%	99%
PaO2 arterial blood gas in room air	115 mmHg	NA	50 mmHg	35 mmHg	NA
A-a gradient in room air	-6 mmHg	NA	61 mmHg	75 mmHg	NA
White blood count	8400/uL (5500-15,500)	3000/uL (4500-13,000)	1390/uL (4000-10,000)	1500/uL (4000-10,000)	4000/uL (4000-10,000)
Neutrophils	2200/uL (1500-9000)	1000/uL (1800-8000)	400/uL (2500-7800)	400/uL (2500-7800)	1400/uL (2500-7800)
Hemoglobin	13.9 g/dL (11.5-13.5)	10.9 g/dL (12-16)	9.4 g/dL (12-16)	10.4 g/dL (12-16)	16 g/dL (12-16)
Platelets	307,000/uL (100000-450,000)	48,000/uL (150000-450,000)	23,000/uL (150000-450,000)	19,000/uL (150000-450,000)	153,000/uL (150000-450,000)
Lymphocytes	4700/uL (1700-6900)	1700/uL (1200-3600)	840/uL (1200-3600)	800/uL (1200-3600)	1290/uL (1200-3600)
T cells (CD3+)	NA	NA	768/uL (800-3500)	NA	1060/uL (798-2823)
B cells (CD19+)	NA	NA	49/uL (200-600)	NA	120/uL (82-476)
NK cells (CD56+)	NA	NA	22/uL (70-1200)	NA	79/uL (66-745)
IgG	NA	15.60 g/L (5.30-13)	14.60 g/L (5.76-12.65)	12.20 g/L (7.51-15.60)	9.10 g/L (7.51-15.60)
IgA	NA	3.23 g/L (0.60-2.70)	4.11 g/L (0.81-2.32)	4.10 g/L (0.82-4.53)	3.19 g/L (0.82-4.53)
IgM	NA	0.85 g/L (0.43 - 1.73)	0.67 g/L (0.30-1.59)	1.13 g/L (0.46-3.04)	0.47 g/L (0.46-3.04)
AST	166 U/L (5-37)	46 U/L (≤ 32)	32 U/L (≤ 32)	27 U/L (≤ 32)	23 U/L (≤ 31)
ALT	187 U/L (5-37)	15 U/L (≤ 31)	20 U/L(≤ 31)	19 U/L (≤ 31)	14 U/L (≤ 31)
Gamma GT	27 U/L (7-32)	69 U/L (≤ 35)	13 U/L (≤ 35)	13 U/L (≤ 35)	7 U/L (≤ 40)
Total bilirubin	0.43 mg/dL (0.2-1)	1.58 mg/dL (≤ 1)	3.79 mg/dL (≤ 1)	5.17 mg/dL (≤ 1)	0.38 mg/dL (≤ 1.18)
INR	NA	1.3	1.5	1.3	1
Albumin	45 g/L (32-52)	38 g/L (35-52)	35 g/L (35-52)	37 g/L (35-52)	39 g/L (35-52)

*A-a* Alveolar-arterial, *ALT* Alanine transaminase, *AST* Aspartate transaminase, *CMV* Cytomegalovirus, *Gamma GT* Gamma-glutamyltransferase, *HPS* Hepatopulmonary syndrome, *Ig* Immunoglobulin, *INR* International normalized ratio, *mmHg* Millimeters of mercury, *NA* No data available, *NK* Natural killer, *uL* Microliter, *%ile* Percentile. Reference ranges for age in brackets

At the age of 15 years she was referred to the pediatric immunologist for recurrent respiratory and skin infections. On physical examination central cyanosis and digital clubbing were prominent, in the absence of other signs suggestive of chronic lung disease. Transcutaneous oxygen saturation at rest was 88-92% in room air. Chest computed tomography (CT) was normal, echocardiography showed normal cardiac anatomy and intracardiac shunt was excluded by cardiac catheterization. HPS was suspected and confirmed by MAA scan, demonstrating a shunt fraction of 38%. HPS worsened, causing severe hypoxemia with SatO2 70-80% at rest, decreasing to 67% on walking. Lowest measured PaO2 was 35 mmHg in room air with an alveolar-arterial gradient (A-a gradient) of 75 mmHg.

**Fig. 1:**
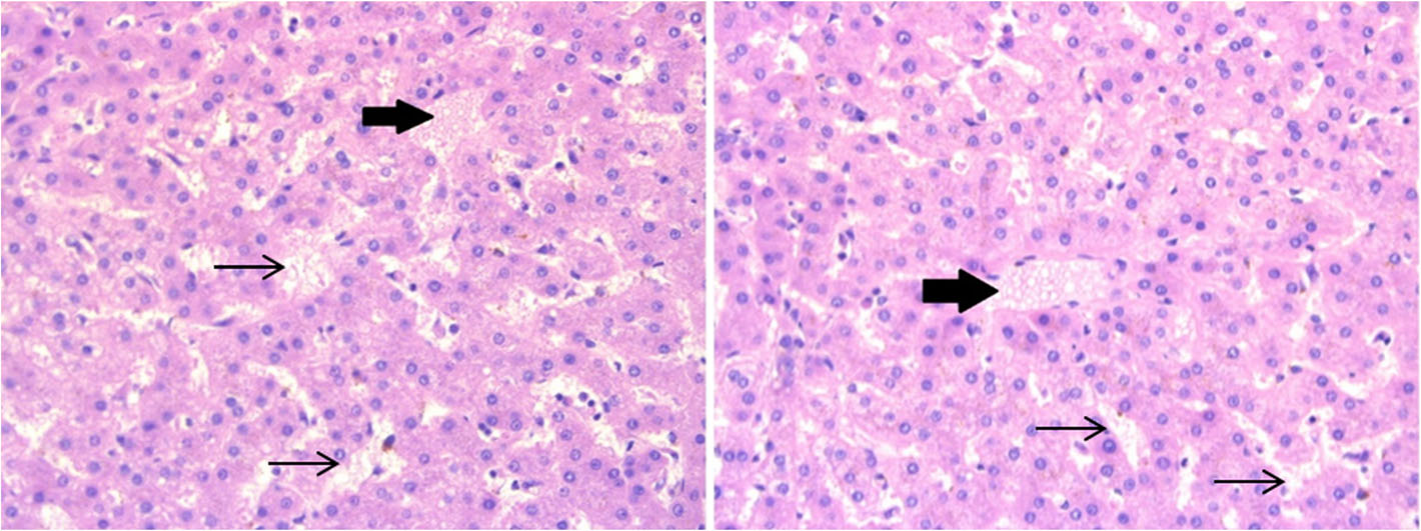
Biopsy of the explanted liver. The hematoxylin and eosin stain shows hyperplasia of hepatic stellate cells, which have a foamy cytoplasm containing abnormally large fat droplets (big arrows), in the context of enlarged sinusoids (thin arrows)

She was listed for liver transplantation and at the age of 18 years she received a deceased-donor orthotopic liver from a HBV core antibody positive donor. She received an increased MELD score of 22 for HPS, while her lab MELD score was 16 at the time of transplantation. Induction immunosuppressive treatment consisted of methylprednisolone (0.2 mg/kg/day to 0.6 mg/kg/day), basiliximab (20 mg in single doses on day 0 and day 4) and tacrolimus (trough levels aimed at 7-10 ng/mL). She received prophylactic anti-HBV immunoglobulins and lamivudine. Perioperative G-CSF treatment and antibiotic and antifungal prophylaxis were added in the context of immunodeficiency. The post-operative recovery was complicated by a hepatic artery stenosis, which was treated by stenting. A few days after transplant complete resolution of hypoxemia, with return to normal SatO2 levels (95-99% in room air) at rest and on exercise within 5 weeks from transplantation. At present, four years after liver transplant, the patient has an excellent quality of life. She is on tacrolimus (trough levels aimed at 3-5 ng/mL) and prophylactic lamivudine. The explant liver histopathology confirmed incomplete septal cirrhosis, with anomalies in the microcirculation and persistent hyperplasia of the hepatic stellate cells, containing abnormal fat droplets (Fig. 1).

Hematopoietic stem cell transplantation was not considered for this patient, in the absence of strong indications such as myelodysplastic syndrome, for which she underwent yearly bone marrow examinations. Moreover, the cause of liver transplant was vitamin A intoxication, and therefore there was no risk of a relapse of liver disease connected with the underlying SDS after transplantation.

## Discussion

We describe a patient with SDS who successfully underwent liver transplantation for portal hypertension with HPS due to vitamin A intoxication. The outcome was excellent despite the presence of immunodeficiency and the severity of the HPS.

Chronic use of high doses of vitamin A (usually > 40,000 IU daily for years) or excessively high doses over a short period (usually > 100,000-200,000 IU daily for days/weeks) invariably lead to liver damage, that can be reversible or not depending on the length and amount of exposure, individual susceptibility and the presence of other health conditions [4, 5]. Vitamin A intoxication usually arises from vitamin A supplement abuse, more rarely from very high dietary intake. Around 90% of total body vitamin A is stored in the liver, where it is found predominantly in the hepatic stellate cells (79-84%) [4]. As a result, vitamin A measurements in blood do not reflect the amount of accumulation in the liver and are therefore unreliable, hence the blood vitamin A levels in this and other reported patients always remained in the normal range despite proven extensive hepatic stellate cells hyperplasia and liver damage [9, 10]. Some reports suggest that serum total retinyl esters measurement represents a more reliable assessment of total body vitamin A content and intoxication [9, 10]. Others propose isotope dilution testing with deuterated or 13C-labelled retinyl acetate tracer, as the only precise estimate of liver vitamin A reserves [11]. This test is however very expensive and not routinely available. Currently, there is no reliable marker for optimal dosing of vitamin A.

Children are more susceptible than adults to vitamin A intoxication, with some reports of toxicity with less than 2000 IU/kg per day. This entails very narrow margins between vitamin A recommended daily allowances and the tolerable upper limit of intake. Most cases of vitamin A intoxication in adults are reported with chronic intake of 50,000 IU daily [4, 5]. This case is exceptional as the patient had exocrine pancreatic insufficiency and therefore fat malabsorption, for which vitamin A substitution is common practice based on the assumption that there will be deficiency of fat-soluble vitamins. Liver disease is associated with SDS but is usually reported as benign hepatomegaly and/or asymptomatic elevation of transaminases with spontaneous resolution, even though severe disease has been reported, such as cholestatic liver disease and fibrosis, while steatosis or hepatic stellate cells hyperplasia have not been described in patients with SDS and liver disease [3, 8]. In this girl clinical and histopathological liver disease were present at 2 years of age; in conjunction with vitamin A intoxication, it progressed to portal hypertension and secondary HPS.

The histopathological finding associated with vitamin A-induced liver disease is characterized by pathognomonic hepatic stellate cells hyperplasia, with the presence of large and numerous lipid-filled vacuoles in the cell cytoplasm under electron microscopy and the obliteration of the space of Disse with collagen deposits, known to give rise to portal hypertension [5]. The histological similarity with primary biliary cirrhosis has led to the recommendation of ursodeoxycholic acid as a potential treatment for vitamin A-induced liver disease [12]. Our patient had mild cholestatic anomalies on initial liver biopsy and had received first prophylactic and then high amounts of vitamin A supplementation for 10 years before portal hypertension was diagnosed. The mild liver disease often observed in SDS was probably a prerequisite for vitamin A induced chronic liver damage to ensue.

Patients with a primary immunodeficiency prove to be challenging in the context of solid organ transplantation. First, they usually have an underlying higher risk of infections, which is augmented by the immunosuppression necessary for the transplant [13]. Second, the defective immune system, that one would intuitively think helpful in lowering the risk of rejection, may in fact predispose them to graft-versus-host disease (GVHD) [14]. This often-fatal complication of transplantation arises from the attack performed on the recipient’s tissues by the donor’s T lymphocytes present in the graft. Lymphoid cells are present in hilar lymph nodes and in the liver parenchyma. On average a liver contains around 10^10^ lymphocytes, which corresponds to approximately 100-200 × 10^6^ lymphocytes/kg for a recipient of liver graft (an allogeneic bone marrow transplantation contains around 30-60 × 10^6^ lymphocytes/kg) [15]. However, GVHD only happens if the histo-incompatibility between donor and host is significant and if the host doesn’t have the capability of mounting a sufficient immune response against the graft, as is especially the case for T cell immunodeficient patients [14]. Despite these concerns, this paper illustrates that liver transplantation is possible in patients with primary immunodeficiency. Judicious choice of induction therapy, maintenance immunosuppression (with therapeutic drug monitoring) and the necessary anti-microbial prophylaxis or direct treatment of the immune deficiency (e.g. G-CSF in this case) are however important [13]. A word of caution is needed though, as in some disorders correction of the underlying immune defect by hematopoietic stem cell transplantation (HSCT) is essential in combination with solid organ transplantation [16]. For this reason, a thorough work-up of the patient in view of a possible HSCT should be performed before a solid organ transplant, and the search of potential stem cells donors should be started when required,

Finally, our patient had severe HPS, with a PaO2 of 35 mmHg on arterial blood gas analysis, A-a gradient of 75 mmHg and a severe intrapulmonary shunting with a MMA shunt fraction of 38% on lung nuclear scanning, fitting the criteria for very severe HPS [6]. She successfully underwent liver transplantation with a complete and swift recovery of both liver and pulmonary function.

In conclusion, we report a case of successful liver transplantation in a patient with a syndromic immunodeficiency (SDS). There are three important lessons to be learned from this case. First, chronic administration of vitamin A entails an important risk of vitamin A intoxication, particularly in children and especially if an underlying condition predisposes to liver disease. Second, serum vitamin A levels are not reliable for monitoring potential intoxication. Third, an underlying primary immunodeficiency is not necessarily a contraindication for solid organ transplantation. Finally, in accordance with recent reports [6, 7] and our own experience, very severe HPS should not exclude patients from liver transplantation.

## Abbreviations

A-a gradient: Alveolar-arterial gradient
G-CSF: Granulocyte colony stimulating factor
GVHD: Graft-versus-host disease
HPS: Hepatopulmonary syndrome
IPVD: Intrapulmonary vasodilation
MDS: Myelodysplastic syndrome
MMA scan: Macro-aggregated albumin scan
PaO2: Partial pressure of oxygen in arterial blood
SatO2: Oxygen saturation
SDBS: Shwachman-Diamond-Bodian Syndrome gene
SDS: Shwachman-Diamond syndrome
